# Supplementary material for: Amblyopia risk factors among pediatric patients in a hospital-based setting using photoscreening
Source: PLoS One. 2021 Jul 29;16(7):e0254831. doi: 10.1371/journal.pone.0254831 (PMC8320995; doi:10.1371/journal.pone.0254831)
Supplement: Attachment — Submitted filename: Response to Reviewers.docx [file pone.0254831.s002.docx]

Apr 1, 2021

Dear Dr Chenette and PLOS ONE Editorial Board,

Thank you for your consideration of our manuscript entitled "Refractive Errors among Pediatric Patients in a Hospital-Based Setting Using Photoscreening". We appreciate the time and effort that has gone into reviewing our manuscript, and thank you for the valuable comments and recommendations.

Please find below a point-by-point reply to the editor and reviewer comments. Comments are copied verbatim, followed immediately by our response.

ACADEMIC EDITOR:

The manuscript has many fundamental issues that need meticulous revision.

1- The manuscript needs English editing for grammar and style

We have revised thoroughly the whole manuscript for grammatical and linguistic errors/ style.

2- Some sections such as the introduction section are too long and contain unnecessary data while the results section needs more details

The introduction was made more succinct and only relevant data were kept. The results section was elaborated more on.

3- The fact that refractive errors were diagnosed with photoscreening rather than a more accurate method like retinoscopy or conventional autorefractors should be addressed. The authors might consider using the term "detection of amblyopia risk factors" rather than "refractive errors"

Thank you for the comment, the authors agree and have modified the title and manuscript text to use “amblyopia risk factors” rather than “refractive errors.”

Reviewer #1: I have one fundamental objection against methodology of this study: I do not agree that photoscreener can be used for evaluation of refractive error prevalence, and this is also showed by the results of this study. It is however accepted that it is used in screening of amblyopia risk factors. Because of that the whole study, to be accepted for publication, must be completely re-written. Moreover, I do not think that referral rate is so much interesting as long as you did not analyze also all negative cases. However, verification with complete ophthalmic examination and cycloplegia refraction of positive cases is very interesting issue and could a subject for a separate article in the future.

We thank the reviewer. We have modified accordingly the title to “Amblyopia Risk Factors among Pediatric Patients in a Hospital-Based Setting Using Photoscreening;” additionally, we revised the manuscript text to reflect our aim as detection of amblyopia risk factors in children visiting our medical center rather than prevalence of refractive errors. We agree that reporting the validation of photoscreening positive results by cycloplegic refraction will be an interesting topic in a future study.

Reviewer #2: This study shows the results of hospital-based screening of Lebanese pediatric patients 2-6 years regarding amblyogenic refractive errors and the results of subsequent comprehensive ophthalmic evaluation. There are some concerns regarding methodology and end results.
1. The manuscript needs significant English language editing.

We have revised the whole manuscript for grammatical and linguistic errors.

2. The “INTRODUCTION” section is very confusing and contains lots of unnecessary data. Specifically, the second paragraph should be totally rephrased. The whole “INTRODUCTION” section should be 2 to 3 paragraphs describing in brief background and the aim of the study.

The introduction was made more succinct and relevant points were kept. Specifically paragraph 2 was rephrased and the whole introduction was shortened.

3. The authors did not mention the period of time during which the study was conducted.

The study was conducted over a time span of 2 years (2018-2020), this was added to the methods section.

4. In “RESULTS” section, the authors mentioned that only 59% of their referred cases were examined at their facility, without mentioning where the rest of referred cases (31%) were examined?

The rest of referred cases were lost to follow up after multiple attempts to schedule an ophthalmology appointment. This was now added to the text for clarification.

Reviewer #3: A very nice, respectful and valuable work that is worthy more evaluation with a larger number of patients for a better benefit.

We thank the reviewer for their positive feedback and appreciate their comment, indeed we plan on a larger scale “school-based” prevalence study when time permits.

29 prevalence (would be better than rate)

Thank you, it was corrected accordingly.

73-76 If you would like to rephrase.

The section was rephrased for better clarity to the reader.

129-133 Table 1 AAPOS ARFs (if you would like to mention ; limit of astigmatism 90/180 Vs oblique one ?, type of anisometropia)

Although it has been reported that oblique astigmatism constitutes a greater amblyopia risk factor than regular astigmatism, the guidelines published by the AAPOS Vision Screening Committee (JAAPOS 2013;17:4-8, Table 1: AAPOS ARFs), which we have followed in this study, did not differentiate by the axis of astigmatism, but rather used the magnitude in Diopters. Similarly, for anisometropia, only the absolute value in diopters was used.

140 Was CRx performed by the same examiner?

Yes the same pediatric ophthalmologist (author C Al-Haddad) examined all referred patients, this is now mentioned explicitly in the methods section.

141,142 Source of this rational of cycloplegia ?

Thank you for your comment. In this clinic, we use two sets of dilating drops as a standard especially with darker irides (which constitute a good portion of our population, below references). This was added to our methods section.

References :

Farhood, Q. K. (2012). Cycloplegic refraction in children with cyclopentolate versus atropine. *J Clin Exp Ophthalmol*, *3*(7), 1-6.

Wallace, D. K., Morse, C. L., Melia, M., Sprunger, D. T., Repka, M. X., Lee, K. A., & Christiansen, S. P. (2018). Pediatric eye evaluations preferred practice Pattern®: I. vision screening in the primary care and community setting; II. comprehensive ophthalmic examination. Ophthalmology, 125(1), P184-P227.

174 37/63 were examined at your department. What about the remainder ?

The rest of referred cases were lost to follow up after multiple attempts to schedule an ophthalmology appointment. This was now added to the text.

312 "still be treated" Do you mean amblyopia ttt with refractive adaptation only or in general ?

This was clarified in text: “within the sensitive period of visual development, while still amenable to patch therapy/ refractive adaptation and at an age where amblyopia has not yet become entrenched”
