# Supplementary material for: Amblyopia risk factors among pediatric patients in a hospital-based setting using photoscreening
Source: PLoS One. 2021 Jul 29;16(7):e0254831. doi: 10.1371/journal.pone.0254831 (PMC8320995; doi:10.1371/journal.pone.0254831)
Supplement: Attachment — Submitted filename: Response to Reviewers .docx [file pone.0254831.s003.docx]

June 28, 2021

Dear Dr Chenette and PLOS ONE Editorial Board,

Thank you for your response to our revision on our manuscript entitled "Amblyopia Risk Factors among Pediatric Patients in a Hospital-Based Setting Using Photoscreening". We appreciate the time and effort that has gone into reviewing our manuscript and thank you for the valuable comments.

Please find below a point-by-point reply to the reviewer comments. Comments are copied verbatim, followed immediately by our response.

Reviewer #4:

1- *Abstract: Results "The refractive errors among the referred patients were: 41% astigmatism, 51% hyperopia, and 8% myopia, with no amblyopia detected on eye examination.: Needs rephrasing*

This sentence was rephrased as follows:

The refractive errors observed among the examined patients were distributed as follows: 41% astigmatism, 51% hyperopia, and 8% myopia; amblyopia was not detected.

*2- Add reference to Table 1*

Reference was added to the table title.

*3- Methods: “for verbal children; anterior segment examination; motility examination for any eye misalignment; cycloplegic manual retinoscopy to detect refractive errors (30 minutes after pupillary dilation with Mydriacyl 1% and Cyclopentolate 1%, applied twice 10 minutes apart, in this clinic, we use two sets as a standard especially with darker irides); The sentence needs grammatical revision. It also starts with small letter*

We agree that this was a long run-on sentence. It was reworded as follows:

This entailed age-dependent visual acuity testing: “central steady & maintained” testing for preverbal children and vision charts (Allen pictures and Early Treatment Diabetic Retinopathy Study charts (EDTRS)) for verbal children. Anterior segment examination, motility examination for any eye misalignment, and posterior segment examination using indirect ophthalmoscopy were performed. Cycloplegic manual retinoscopy was done to detect refractive errors (30 minutes after pupillary dilation with Mydriacyl 1% and Cyclopentolate 1%, applied twice 10 minutes apart; in this clinic, we use two sets as a standard especially with darker irides).
*4- Results: "Out of the 37 examined children, 19 had hyperopia, 15 had astigmatism and 3 subjects were myopic." So do this means that none of the patients had both spherical and cylindrical error at the same time?*

Thank you for your comment. As we mentioned in the Methods section:

“Significant astigmatism was defined as cylindrical power of 1D or more with the main refractive error being the cylinder. Those reported with hyperopia and myopia had mainly spherical refractive error (>+1D or <-1D, respectively).”

Thus, the patients were subdivided based on the *predominant refractive error detected* when both spherical and cylindrical errors co-existed.

*5- While correlation was done to compare the readings of autorefractor and retinoscopy, the correct statistical analysis would to use an agreement statistics e.g. Bland-Altman plot and Deming regression analysis*

Thank you for your comment. We agree that an agreement statistic would strengthen our study. We thus added a Bland-Altman plot to our paper.
